# Examining the Effects of Academic Stress, Self‐Efficacy, Cognitive–Behavioral Outcomes, Psychological Distress, and Prosocial Behavior: A Moderated‐Mediation Model

**DOI:** 10.1002/brb3.70907

**Published:** 2025-10-24

**Authors:** Wang Chongjin, Iftikhar Ahmed Charan, Shazia Soomro

**Affiliations:** ^1^ School of Public Administration of Shandong Technology and Business University Yantai Shandong Province PR China; ^2^ Department of Sociology School of Sociology and Political Science of Anhui University Hefei Anhui Province PR China

**Keywords:** academic stress, cognitive behavioral, prosocial behavior, psychological distress, self‐efficacy

## Abstract

**Background:**

Academic stress significantly affects students’ well‐being and academic performance. This study addresses this gap by exploring how academic self‐efficacy mediates the relationship between academic stress and both educational and psychological distress while considering cognitive and prosocial behaviors.

**Methods:**

This study surveyed 412 participants (mean age 22–25 years; 55.1% male, 44.9% female). Descriptive analysis and regression found factors that predict prosocial behavior, and a moderated‐mediation model looked at how academic self‐efficacy affects the link between academic stress and prosocial behavior, with psychological distress and cognitive behavior acting as middle factors.

**Results:**

The mean academic stress score was 3.42 (SD = 0.81), and the mean academic self‐efficacy score was 3.91 (SD = 0.65). Academic stress was negatively correlated with both academic self‐efficacy (*r* = −0.36, *p* < 0.01) and prosocial behavior (*r* = −0.29, *p* < 0.01). Academic self‐efficacy positively predicted prosocial behavior (*b* = 0.35, *p* = 0.001), while academic stress negatively predicted it (*b* = −0.18, *p* = 0.011). Moderated‐mediation analysis revealed that academic self‐efficacy moderated the relationship between academic stress and prosocial behavior (*b* = −0.19, *p* = 0.001). The model explained 60% of the variance in prosocial behavior (*R* ^2^ = 0.60, *p* = 0.001).

**Conclusion:**

This study found that academic stress negatively affects prosocial behavior, while academic self‐efficacy positively predicts it. Self‐efficacy also moderated the impact of stress, suggesting its protective role. The model explained a significant portion of the variance in prosocial behavior, highlighting the importance of enhancing self‐efficacy to mitigate academic stress.

## Graphical abstract

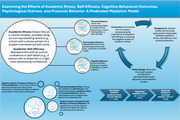

## 1 Background

University students frequently deal with a range of personal, social, and academic difficulties that can have a significant impact on both their academic performance and mental health (Abdelrahman et al. 2025). Students may become especially susceptible to stress and burnout as a result of the shift to higher education, increased academic obligations, financial worries, and social adaptations (Pascoe et al. 2020). The term “academic resilience” describes a student's capacity to manage stress, anxiety, and academic disappointments while still producing successful academic results. It is thought to be crucial for success in postsecondary education (Martin and Marsh 2006). Academic resilience, as described by Cassidy (2016), is the capacity to sustain high levels of motivation and performance in the face of academic challenges (Cassidy 2016). The practice entails employing behavioral, emotional, and cognitive techniques to stay involved and goal‐focused while managing personal challenges, resource constraints, and academic demands (Martin 2013). Morales (2008) asserts that resilient students often support their academic performance and exhibit adversity. Academic resilience has been highlighted in recent research as a protective factor for students' mental health and psychological well‐being, in addition to being a factor in academic accomplishment (Demir 2023; Yang and Wang 2022).

Academic stress and academic self‐efficacy are complex issues that have a significant impact on university students' academic performance and general well‐being worldwide. This stress extends beyond the classroom, influencing various aspects of students’ personal lives. Many students suffer from physical symptoms, including fatigue, sleep difficulties, and a chronic sense of exhaustion, in addition to psychological repercussions like anxiety and depression (Pérez‐Jorge et al. 2025). The pandemic has significantly impacted academic environments across different educational systems. Using Barraza's SISCO inventory of academic stress, a descriptive study carried out in Ecuador discovered that one of the main causes of students' stress was task overload (Moreno‐Montero et al. 2022; Pérez‐Jorge et al. 2021). If not addressed appropriately, this stress can result in burnout, which is characterized by extreme fatigue along with physical and psychological negative effects (Escuderos et al. 2017).

Perceived stress is how individuals personally perceive the unpredictability, lack of control, or overwhelming nature of their circumstances, especially when they feel external pressures are greater than their ability to cope (Cohen et al. 1983). In contrast to external stressors, it centers on how a person personally evaluates and emotionally reacts to demanding situations, giving it unique weight in shaping psychological outcomes (Abdelrahman et al. 2025). Within universities, elevated levels of perceived stress typically correlate with lower grades, reduced well‐being, and diminished resilience among students (Ribeiro et al. 2018; Slimmen et al. 2022). Students are especially vulnerable to this form of stress because they juggle heavy course loads, shifting social norms, financial constraints, and major life transitions (Pascoe et al. 2020). For example, academic and environmental stressors are the main causes of moderately high levels of stress that university students frequently experience (Alkhawaldeh et al. 2023).

Recent studies show that university students are feeling more academic stress and anxiety since classes moved online. The quick switch to virtual learning, combined with losing regular social contact during and after the pandemic, hit American students hard, causing sharper levels of study‐related stress, anxiety, and even depressive symptoms (Son et al. 2020). A similar look at New Jersey undergraduates found that pressure from schoolwork and worries about the future ranked as the main drivers of serious mental health problems (Kecojevic et al. 2020). Gavurova et al. (2022) found that the shift to virtual learning increased time on digital devices and that excessive screen use worsened symptoms of depression, anxiety, stress, and internet addiction (Gavurova et al. 2022). Lee et al. (2021) also examined university students during the pandemic; they reported that rising levels of stress, anxiety, and depression drove more students to seek mental health services, signaling a growing need for psychological support on campus (Lee et al. 2021). In a study by Martínez‐Líbano et al. (2023), the long‐term impact of postpandemic higher education on young adults’ mental health was examined. Their findings show that stress, anxiety, and depression constantly affect students, which is consistent with the results of this study (Martínez‐Líbano et al. 2023). These findings highlight how psychological and social interventions are still necessary to aid in students' recovery today.

Research consistently finds that high levels of academic stress and low academic self‐efficacy predict damaging outcomes such as unfinished assignments, withdrawn courses, and lowered grades (Misra and Castillo 2004). Students facing intense pressure often report physical, symptoms, including chronic fatigue, diminished appetite, headaches, and gastrointestinal discomfort (Winkelman and Shapiro 1994). Beyond these issues, persistent stress in the classroom also raises the likelihood of substance use and even aggressive behaviors. While researchers have extensively documented the negative effects of academic stress on college students' mental health, they have begun to consider whether the same pressure can also propel positive social outcomes, including prosocial behaviors and civic engagement (Cruz 2024). Exploring both sides offers a more balanced view of how campus life shapes young adults' moral and social development. The impact of academic stress on various forms of prosocial behavior may differ depending on the behavior's characteristics.

**FIGURE 1:**
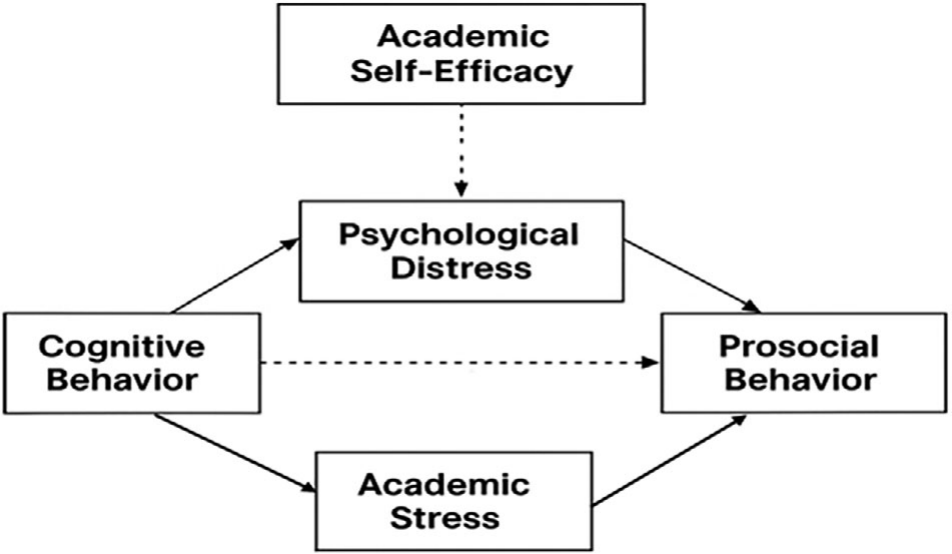
Conceptual framework.

Prosocial behavior directed at in‐group members usually builds on established bonds and the wish to keep these connections upbeat, so high academic stress is likely to change such actions only a little (Khanehkeshi 2011; Misra and McKean 2000; Sharma and Passi 2022). In contrast, helping people outside one's close circle or participating in community projects often requires individuals to set aside their needs and make room for others, a skill that heavy study loads can deplete (Mesurado and Richaud 2017). Earlier research shows that when strangers are involved, moral identity, social rank, broad values, accepted norms, and empathy matter most; the personal history that guides in‐group help becomes far less relevant (Carlo and Padilla‐Walker 2020). Furthermore, for civic engagement to be successful, preparation and self‐control are necessary (Davis et al. 2020; Pastorelli et al. 2021). This research investigates the effects of academic stress and interplay factors such as academic self‐efficacy, cognitive behavior, and psychological distress, with a focus on how prosocial behavior influences these connections among young adults. The goal is to offer both theoretical and empirical insights into the challenges of academic stress and anxiety and provide strategies for addressing these issues. Figure 1 presents the conceptual framework illustrating the relationship among cognitive behavior, academic stress, psychological distress, and prosocial behavior, with academic self‐efficacy moderating key pathways.

## 2 Literature Review

### 2.1 Academic Stress and Academic Self‐Efficacy

Research indicates that insufficient academic preparation, misguided self‐perception, and a marked absence of motivation can combine to push students toward academic burnout (Gao 2023). Academic stress continues to plague many young adults on university campuses and beyond, often occupying center stage in their cumulative stress profile (Nagamitsu et al. 2020; Ye et al. 2019). Stress, when in modest doses, can sharpen concentration and enhance productivity (Henderson et al. 2012; Lin et al. 2022). However, when the pressure intensifies, constructive motivation quickly transforms into negative feelings about classes (Kumari 2020), slipping grades (Savarese et al. 2019), and self‐defeating habits such as procrastination (Niazov et al. 2022) and habitual smartphone use (Shen et al. 2021). These maladaptive behaviors undermine academic performance and pave the way for deeper emotional disturbances, including symptoms of depression and even suicidal ideation. Recent evidence suggests that elevated levels of exam‐related anxiety and workload pressure may themselves act as early warning signs of broader mental health decline (Gao 2023). In short, the weight of academic demands shapes young adults’ cognitive, behavioral, and emotional development, for better or worse. As a result, academic stress affects young adults' emotional, behavioral, and cognitive development. Problem behavior theory states that both internal personal characteristics and external environmental perceptions can impact behaviors that can result in academic stress. Students' conduct can be directly impacted and stress levels raised when they feel academic stress. The transactional model of stress and coping proposes that people evaluate a potential stressor in two stages, first by deciding whether it is harmful and then by weighing their resources to deal with it (Lazarus 1984), if students judge exam pressure or heavy workloads as a genuine threat, this belief often guides their emotional and behavioral reactions and, in many cases, leaves them feeling defeated and overwhelmed (Walburg 2014). Such negative assessments not only drain energy in the short term but, over time, accumulate and help trigger the deeper phenomenon of academic burnout, in which motivation, creativity, and even basic enjoyment of study disappear (Gonzálvez et al. 2018).

Academic self‐efficacy reflects a person's belief in their ability to accomplish or achieve academic tasks at different stages of their educational path (Schunk 1991). It reflects the level of confidence a person has in achieving their academic goals (Weißenfels et al. 2023). According to the processing efficiency theory, self‐regulation and control mechanisms may have an impact on the association between anxiety levels and performance, even if academic anxiety can affect academic burnout in middle school pupils. In the academic context, scholars suggest that students’ confidence in their abilities, academic self‐efficacy, may moderate the link between school‐related stress and feelings of anxiety (Eysenck and Calvo 1992; Michael 1982). When learners believe they can perform well, they tend to approach stressful tasks with calmer thoughts and steadier actions. Bandura's well‐known theory of self‐efficacy supports this idea, arguing that expectations about personal experience color every form of coping that follows an external stress (Bandura 1977). Thus, students who genuinely trust their study skills often avoid the worst emotional consequences that pressure can bring. Supporting this claim, recent research on Chinese undergraduates shows that high self‐efficacy weakens the tie between stress and negative moods such as sadness, anxiety, and even full‐blown depression (Schönfeld et al. 2019).

Additionally, studies on the relationship between academic self‐efficacy and life satisfaction have shown that when faced with stress and anxiety, young adults who have higher academic self‐efficacy report higher levels of life satisfaction, whereas those who have lower self‐efficacy exhibit more significant distress (Gao 2023). Scholars argue that academic self‐efficacy serves as a second‐level appraisal for young adults when they confront school‐related academic stress, building on the transactional model of stress and coping proposed by Lazarus (1984). When students doubt their abilities, they are more likely to view these pressures as genuine threats; that elevated threat perception is strongly linked to higher academic burnout rates. From this perspective, self‐efficacy partially protects learners by reducing the impact of academic stress on anxiety, thereby moderating rather than eliminating the relationship between the two.

**H1**: Academic stress mediates the relationship between cognitive behavior and prosocial behavior.
**H2**: Academic self‐efficacy moderates the relationship between academic stress and psychological distress, such that the relationship is weaker at higher levels of academic self‐efficacy.
**H3**: Academic self‐efficacy moderates the relationship between psychological distress and prosocial behavior, such that the negative relationship is weaker at higher levels of academic self‐efficacy.

### 2.2 Psychological Distress and Cognitive Behavioral

Students’ mental health is significantly impacted by common psychological illnesses such as anxiety, cognitive–behavioral disorders, and psychological discomfort, which present as a range of emotional, behavioral, and cognitive symptoms. The significant influence this illness has on students' academic and personal lives is highlighted by the fact that they commonly express these symptoms as concern and anxiety (Nwadi et al. 2025). A particular form of anxiety, often called test anxiety, appears most clearly during formal examinations and routinely undermines students’ grades. This reaction has little to do with actual intelligence; rather, it exposes the crushing tension that peak evaluation moments can produce (Nwadi et al. 2025; Torrano et al. 2020). Test‐related stress comprises both emotional turmoil and measurable physical signs, manifesting before, during, or after the exam and steering academic performance in the wrong direction. According to Jarso et al. (2024), students who suffer from high levels of anxiety frequently have trouble processing and remembering information during tests because of their negative thought patterns and ongoing concerns (Jarso et al. 2024).

Test anxiety consists of a cognitive–behavioral cycle in which persistent worry and negative self‐talk feed one another; at the same time, the emotional side can show up physically in tension, shaking, and a racing heart. Because stress clouds thinking, students frequently postpone studying or rely on last‐minute cramming, which only deepens the anxiety that drove the poor habits in the first place (Jarso et al. 2024). Academic stress has been shown to negatively impact mental and physical health, frequently affecting sleep, social interactions, and even part‐time employment (Pérez‐Jorge et al. 2025). From a cognitive–behavioral viewpoint, overriding academic stress erodes attention, undercuts memory retrieval, and saps the self‐confidence energetic for performance, sometimes translating directly into failing grades or lost opportunities. In parallel, the body registers the stress: hormone levels swing, sleep becomes elusive, and weight creeps up or down (Peláez et al. 2021). High‐pressure moments, such as examination weeks, prompt many students to fall back on unhealthy routines, eating more processed food, relying on stimulants or depressants, and moving their bodies even less, thereby intensifying risk factors for dyslipidemia, anxiety, and clinical depression (Eusebio 2016; Hickie et al. 1995).

Recent work shows that students' stress levels do not arise from a single cause; instead, they emerge from the way psychological distress, academic stress, and a sense of academic self‐efficacy interact with one another (Kristensen et al. 2023). The authors stress that lifting self‐efficacy should form a key part of any coping plan aimed at reducing overwhelm. They also point out that strong peer ties, supportive family relations, and responsive academic networks can shield students from anxiety, making pupils more engaged in their work (Chaudhry et al. 2024). Beyond practices such as mindfulness and deep breathing, effective management of school‐related stress typically combines problem‐focused strategies, fixing the source of worry, and emotion‐focused approaches, regulating feelings as obstacles arise. The adverse impacts of stress on students' academic performance and overall health, behavior, and psychological well‐being will be mitigated as they manage diverse stressors. Enhancing emotional intelligence and relying on a support network will be crucial (Gavurova et al. 2022; Tsantopoulos et al. 2022). According to the Sustainable Development Goals, providing students with stress management skills and creating an environment in the classroom that supports their well‐being are essential for both their academic achievement and personal development (Restrepo et al. 2020).

**H4**: Cognitive behavior is positively associated with prosocial behavior.
**H5**: Psychological distress mediates the relationship between cognitive behavior and prosocial behavior.

### 2.3 Prosocial Behavior

Studies have demonstrated that several forms of stress can affect civic involvement and prosocial behaviors, but few have looked at the connection between academic stress and prosocial behavior. Some studies suggest that economic stress may impact prosocial behaviors in a variety of ways (Cruz et al. 2025). According to one study, prosocial activities driven by selflessness were positively correlated with economic stress, while prosocial behaviors driven by selfishness were adversely correlated (Davis et al. 2017). Recent investigations have begun to map how economic stress influences civic engagement and both in‐group and out‐group helping behaviors among emerging adults. While academic pressure showed little direct link to any form of prosocial action, financial stress was anticipated to be greater in‐group assistance among students reporting robust community self‐efficacy (Davis et al. 2022). The present study, therefore, asks whether similar patterns emerge when academic stress, rather than economic strain, is considered, and how outcomes relate to self‐efficacy. It also tests whether cognitive coping strategies and levels of psychological distress can buffer the negative impact of academic stress and anxiety on prosocial engagement.

Prosocial behaviors contribute meaningfully to academic achievement, temper self‐centeredness, and nurture constructive exchanges with peers, family members, instructors, and friends. Attending to another person's needs and providing support during challenging moments proves advantageous to both the helper and the recipient (Petruța and Stănculescu 2025). This has led researchers to focus on how prosocial behavior can be encouraged in educational environments. However, few studies have explored the psychological benefits of prosocial behavior during early adulthood (Caprara et al. 2014; Hardy and Carlo 2005; Oberle et al. 2023). An important behavioral outcome when considering the impact of stress on young adults. Additionally, prosocial behaviors play a significant role in reducing students' stress by involving actions such as helping friends and benefiting others, which can include offering comfort, donating time, volunteering for activities, or providing resources (Carlo and Randall 2002; Eisenberg and Miller 1987). These behaviors are seen as indicators of positive personal development. Most importantly, prosocial behaviors help strengthen social interactions through experiences. Prosocial behaviors are directed toward individuals, such as friends or strangers, while others are aimed at the broader community. Civic engagement, which involves participation in community activities, is a key form of prosocial behavior. It reflects an individual's awareness of and involvement in efforts to improve the social or environmental landscape (Carlo 2006; Carlo and Randall 2002; Sze‐Yeung Lai and Chi‐leung Hui 2021).

## 3 Materials and Methods

### 3.1 Design

This study utilized a moderated‐mediation model to examine the effects of academic stress, academic self‐efficacy, cognitive behavior, psychological distress, and prosocial behavior as moderating factors. The objective was to investigate how these variables interact with perceived stress in different academic settings.

### 3.2 Settings

We obtained a representative sample using a multistage random cluster sampling technique. The sample size was determined using a very rough guideline of having about 20–35 majors for every different parameter to be estimated in the model (Kline 2023). For this study, we selected young adults enrolled in various majors based on the nature of the research questions. To meet the research objectives, we conducted a survey and used whole‐group sampling. We selected a representative group of undergraduate and postgraduate students from universities in Sindh province in Pakistan to gather the necessary data. All recruitment materials included the primary researcher's email address, and prospective participants were invited to reach out, set up a meeting, and fill out the survey. Those who agreed were reminded that taking part was completely optional, that they could leave any question unanswered, and that they could stop participating at any point prior to wrapping up the survey. Each participant received a copy of the consent form before the study began, and the research team explained the main goals of the project in plain language, providing immediate answers to any questions. The investigation conformed to the ethical principles outlined in the Helsinki Declaration. During these initial discussions, participants were reminded that their answers would remain confidential, that joining the study was entirely voluntary, and that they could withdraw at any time without penalty. Written consent from all individuals was collected and secured before any data were recorded.

### 3.3 Participants

To obtain a more thorough understanding of academic stress and its relationship to various educational levels, this study included both undergraduate and postgraduate students. These groups were chosen because, despite being at different points in their educational paths, both face considerable pressures. Undergraduates typically deal with the challenges of adapting to higher education and handling coursework, while postgraduates often experience the stress of conducting advanced research, completing theses, and juggling academic duties with professional commitments (Pérez‐Jorge et al. 2025). Participants were chosen through straightforward random sampling procedures. A total of 412 students (mean age 22–25 years; range 18–25 years) were invited to complete the online questionnaire, and 450 usable surveys were ultimately returned, yielding a valid response rate of 91.5%. Among the participants, 227 (55.1%) were male and 185 (44.9%) were female. The sample consisted of 247 undergraduate (60.0%) and 165 postgraduate (40.0%) students with ages ranging from 18 to 30 years (average age = 20.2 ± 0.77). The young adults in the study came from urban 288 (70.0%) and rural 124 (30.0%) areas, ensuring that the sample accurately represented the broader young population (Table 1).

**TABLE 1:** Demographic information of participants (*N* = 412).

Variable	Category	Frequency (*n*)	Percentage (%)
**Gender**	Male	227	55.1%
	Female	185	44.9%
**Age group**	18–21 years	144	35.0%
	22–25 years	196	47.6%
	26–30 years	72	17.5%
**Education level**	Undergraduate	247	60.0%
	Postgraduate	165	40.0%
**Residence**	Urban	288	70.0%
	Rural	124	30.0%
**Relationship status**	Single	319	77.4%
	In a relationship	93	22.6%

### 3.4 Measurements of Academic Stress

The questionnaire was developed to gather detailed sociodemographic information from students, offering a complete profile of the participants. The survey contained items addressing demographic and lifestyle variables, including gender, age, educational level, living situation, current relationship status, prior training in stress and anxiety management, how often participants used such practices, and whether support resources were readily available.

#### 3.4.1 Academic Stress

To assess academic stress at various educational levels, this study utilized a questionnaire designed specifically for university students. Researchers measured students' experiences of stress at different points in the academic year by administering the Scale for Assessing Academic Stress (Sinha et al. 2001). The instrument offers 15 survey items answered with 1–5 “Disagree” to “Strongly Agree,” allowing participants to indicate whether each statement describes their daily lives. Designed to capture a broad spectrum of pressure, the scale taps into emotional, physical, cognitive, psychological, social‐interpersonal, and affective dimensions. Prior work reported excellent test–retest reliability of 0.94 across a 1‐month interval and split‐half reliability of 0.80 for the same measure (Kaphle et al. 2024). Consistent with these findings, the Stress‐Academic Attitudes Scale (SAAS) exhibited strong internal consistency in the present sample, yielding a Cronbach's alpha of 0.90.

#### 3.4.2 Academic Self‐Efficacy

Participants’ academic self‐efficacy was measured using a five‐item scale adapted from the Patterns of Adoptive Learning Scale (PALS; Midgley et al. 2000). This scale evaluates the level of confidence individuals have in their ability to accomplish academic tasks at both bachelor's and master's levels, including both classroom assignments and homework. One sample statement from the scale reads, “Even when a task is difficult, I can grasp the material, and I will put in the effort.” Respondents evaluated each statement with a five‐point Likert scale, ranging from 1 (not confident at all) to 5 (extremely confident). Earlier research reported satisfactory reliability for the scale, yielding Cronbach's alpha scores above 0.78 (Kristensen et al. 2023).

#### 3.4.3 Psychological Distress

A five‐item condensed version of the Norwegian Symptom Check List‐90‐R was used to evaluate the participants, with special attention paid to the anxiety and depression subscales (Tambs and Moum 1993). This assessment is not meant to diagnose depression or anxiety disorder, but serves as a general measure of combined anxiety and depressive symptoms (Tambs and Moum 1993). The tool is not intended to diagnose specific anxiety or depressive disorders; rather, it provides a broad index of overlapping symptoms in both domains (Siqveland et al. 2016). On a scale of 1 (not at all) to 4 (very much), participants were asked to rate the indicate of distress or discomfort they had felt during the previous 14 days. Examples of the items include “academic pressures, feeling worried, sad, depression, and often anxiety, tense, and mental disorders” for anxiety. Previous research has shown the scale to have good reliability, with Cronbach's alpha values greater than 0.86 (Kristensen et al. 2023).

#### 3.4.4 Cognitive Behavioral

The ARS‐30 (Academic Resilience Scale) was designed to evaluate an individual's ability to cope with academic challenges by measuring their cognitive, emotional, and behavioral responses, all of which are key components of academic resilience (Cassidy 2016). It consists of 20 items, which are rated on a 5‐point Likert scale from “unlikely” (1) to likely (5). The four primary dimensions of the scale are adaptive help‐seeking, perseverance, reflection, cognitive–behavioral impact, and emotional negative reactions (Cassidy 2016). The scale has shown high reliability and internal consistency, with Cronbach's alpha values greater than 0.86 (Abdelrahman et al. 2025; Ramezanpour et al. 2019). Bilingual experts evaluated the translations and cultural suitability of the scale to ensure its appropriateness for the study. Their feedback was used to refine the language and enhance the clarity, ensuring the scale's relevance to the diverse population involved in the research.

#### 3.4.5 Prosocial Behavior

The prosocial behavior measure in this study was adopted from a 15‐item questionnaire that investigates the Strengths and Difficulties questionnaire to assess young adults’ prosocial actions (Goodman 2001). The measure aimed to evaluate the frequency and the nature of prosocial behaviors exhibited by students in response to academic stress. Using the same format and response options as those for anxiety and emotional problems, participants rated the following statements: “I help and show kindness to others and take care of classmates during the academic‐related stress.” “I try to be helpful to others who are hurt, sad, upset, stressed, or ill.” I try to be kind to younger children, and I often volunteer to assist parents, classmates, teachers, and other children. To measure this behavior, a seven‐point Likert scale asks respondents how frequently they help, with “Never” scored 1 and “Always” scored 7. The scale shows strong reliability and internal consistency, as demonstrated by a Cronbach's alpha above 0.86, and the wording was revised to focus specifically on prosocial acts directed toward friends (Ray et al. 2025).

### 3.5 Data Analyses

The study used descriptive statistics, correlation analysis, and a moderated‐mediation model to explore how the key variables relate to one another. Direct and indirect links were tested in analyses that were performed using STATA, following a two‐tailed approach and treating *p* values below 0.05 as significant. An independent‐samples *t*‐test and one‐way ANOVA examined whether demographic factors influence prosocial behavior. Means and standard deviations served as the principal descriptive statistics for summarizing the outcomes. To see how academic stress relates to prosocial behavior and moral identity, we calculated Pearson correlation coefficients and compared them with the overall score from the SAAS. We also employed the chi‐square test to assess potential associations involving categorical and continuous variables. Before analysis, all data were standardized, and we drew a bootstrap sample of 50,000 cases to ensure robust, stable parameter estimates. The research examined how SAAS and moral identity, both separately and together, influenced several key outcomes, using Hayes' PROCESS Model 4 to see if there were any moderating effects and Model 15 to look for indirect effects that depended on certain conditions (Hayes 2018; Kaphle et al. 2024; Liu et al. 2024). To evaluate statistical significance, the authors determined whether the bias‐corrected confidence intervals for the focal effects included zero. They also explored the interrelations among self‐reported academic stress, cognitive appraisal, academic self‐efficacy, psychological distress, and prosocial behavior. The analysis uncovered robust correlations across this set of variables; complete findings are detailed in the next section.

## 4 Results

### 4.1 Descriptive Correlation Analyses

The current study used several self‐report surveys to collect information from participants. To check for common method bias in those responses, we ran Harman's single‐factor test as part of a larger factor‐analysis procedure (Zhang et al. 2024, 2025). We conclude that systematic bias attributable to the shared self‐report format is unlikely to have materially influenced the findings. The study sample (Table 1) consisted of 412 undergraduate and postgraduate students, with 55.1% male and 44.9% female, and an average age of around 20.2 years (SD = 3.99). The analysis results showed a significant correlation among the variables. Psychological distress was found to be negatively associated with academic stress and positively related to prosocial behaviors across different educational stages. However, academic stress did not have a significant relationship with prosocial behaviors or cognitive behavioral. Cognitive–behavioral factors were positively correlated with psychological distress, academic self‐efficacy, and prosocial behaviors across various educational stages. Additionally, there was a positive correlation between cognitive–behavioral factors and prosocial behaviors.

### 4.2 Descriptive Statistics and Correlation Analysis

The results presented in Table 2 highlight a significant correlation among the key variables of include academic stress, psychological distress, academic self‐efficacy, cognitive behavioral, and prosocial behavior. Academic stress (*M* = 3.42, SD = 0.81) was negatively correlated with psychological distress (*r* = −0.41, *p* < 0.01), academic self‐efficacy (*r* = −0.36, *p* < 0.01), cognitive behavioral (*r* = −0.33, *p* < 0.01), and prosocial behavior (*r* = −0.29, *p* < 0.01) suggesting that higher academic stress is linked to lower levels of psychological well‐being, reduce self‐efficacy, fewer adoptive cognitive strategies, and less prosocial engagement. Participants experienced a moderate level of psychological distress (*M* = 3.76, SD = 0.73) showed a positive correlation with academic self‐efficacy (*r* = 0.44, *p* < 0.01), cognitive behavior (*r* = 0.39, *p* < 0.01), and prosocial behavior (*r* = 0.47, *p* < 0.01), indicating that higher levels of distress were associated with better self‐efficacy, improved cognitive strategies, and increased prosocial actions.

**TABLE 2:** Descriptive statistics and correlations (*N* = 412).

Variable	*M*	SD	1	2	3	4	5
1. Academic stress	3.42	0.81	—				
2. Psychological distress	3.76	0.73	−0.41**	—			
3. Academic self‐efficacy	3.91	0.65	−0.36**	0.44**	—		
4. Cognitive behavior	3.68	0.69	−0.33**	0.39**	0.46**	—	
5. Prosocial behavior	4.05	0.58	−0.29**	0.47**	0.51**	0.43**	—

*Note*: *p < 0.05, **p < 0.01.

The results suggest that higher levels of psychological distress are associated with increased self‐efficacy, enhanced cognitive strategies, and more prosocial actions. Furthermore, academic self‐efficacy (*M* = 3.91, SD = 0.56) also showed a strong positive correlation with cognitive behavior (*r* = 0.46, *p* < 0.01) and prosocial behavior (*r* = 0.51, *p* < 0.01), highlighting the role of self‐efficacy in fostering both cognitive and prosocial behaviors. Table 2 findings suggest that reducing academic stress and enhancing self‐efficacy may be critical in promoting psychological well‐being and prosocial behavior among students.

### 4.3 Hierarchical Regression Analysis Predicting Prosocial Behavior

The hierarchical regression analysis in Table 3 assesses the predictors of prosocial behavior across three steps. In step 1, cognitive behavior (*β* = 0.27, *p* = 0.001), academic stress (*β* = 0.22, *p* = 0.011), and psychological distress (*β* = 0.31, *p* = 0.001) were significant predictors of prosocial behavior, explaining 36% of the variance (*R* ^2^ = 0.36). Step 2 added academic self‐efficacy as a predictor, which significantly increased the explained variance to 51% (Δ*R* ^2^ = 0.15, *p* < 0.001), with academic self‐efficacy showing a strong positive effect on prosocial behavior (*β* = 0.38, *p* = 0.001). Step 3, the interaction terms between academic self‐efficacy and the other predictors were included, revealing significant moderating effects. Specifically, the interaction of academic stress × academic self‐efficacy (*β* = 0.19, *p* = 0.001), psychological distress × academic self‐efficacy (*β* = 0.17, *p* = 0.001), and cognitive behavior × academic self‐efficacy (*β* = 0.14, *p* = 0.001) were all significant, highlighting that academic self‐efficacy moderates the relationship between academic stress, psychological distress, cognitive behavior, and prosocial behavior. The final model explained 60% of the variance in prosocial behavior (*R* ^2^ = 0.60), indicating that the interaction effects of self‐efficacy were crucial in predicting prosocial outcomes. These findings suggest that enhancing academic self‐efficacy may help mitigate the negative effects of stress and distress on prosocial behavior, reinforcing the importance of self‐efficacy in promoting positive social behaviors among students.

**TABLE 3:** Hierarchical regression analysis predicting prosocial behavior.

Predictor	*B*	SE	*β*	*t*	*p*
**Step 1**					
Cognitive behavior	0.21	0.06	0.27	3.5	0.001
Academic stress	−0.18	0.07	−0.22	−2.57	0.011
Psychological distress	0.25	0.06	0.31	4.17	< 0.001
**Step 2**					
Academic self‐efficacy	0.29	0.05	0.35	5.8	< 0.001
**Step 3**					
Academic stress × academic self‐efficacy	−0.14	0.04	−0.19	−3.5	0.001
Psychological distress × academic self‐efficacy	0.11	0.03	0.17	3.67	< 0.001
Cognitive behavior × academic self‐efficacy	0.09	0.03	0.14	2.9	0.004
**Model summary**					
**Step**	***R* ^2^**	**Δ*R* ^2^**	***F***	***p***	
1	0.36	0.21	22.4	< 0.001	
2	0.51	0.15	29.95	< 0.001	
3	0.6	0.09	33.2	< 0.001	

### 4.4 Sample Slopes Analysis and Moderation of Academic Stress

Table 4 presents the results of a sample slopes analysis that looks at how academic self‐efficacy influences the link between academic stress, psychological discomfort, prosocial behavior, and cognitive behavior. Academic stress, the relationship was significantly negative when academic self‐efficacy was low (*β* = −0.32, *p* = 0.001), indicating that students with low self‐efficacy experienced higher academic stress, leading to lower prosocial behavior. However, when academic self‐efficacy was high, this relationship was insignificant (*β* = −0.07, *p* = 0.319), suggesting that higher self‐efficacy mitigates the negative impact of academic stress. Psychological distress, the relationship was positive at both low (*β* = 0.17, *p* = 0.016) and high (*β* = 0.33, *p* = 0.001) levels of academic self‐efficacy, but the effect was stronger when self‐efficacy was high, highlighting that students with higher self‐efficacy exhibit greater distress but still maintain higher levels of prosocial behavior. Similarly, cognitive behavior, the positive relationship with prosocial behavior, was stronger when academic self‐efficacy was high (*β* = 0.28, *p* = 0.001) compared to when it was low (*β* = 0.15, *p* = 0.014). The findings emphasize the role of academic self‐efficacy in moderating the effects of academic stress and distress. Specifically, they suggest that when students have higher self‐efficacy, they are better able to cope with academic stress, which in turn helps to reduce the negative impacts of stress on their prosocial behavior.

**TABLE 4:** Simple slopes analysis for moderator.

Predictor	Level of moderator	*B*	SE	*t*	*p*
Academic stress	Low academic self‐efficacy (−1 SD)	−0.32	0.08	−4	< 0.001
	High academic self‐efficacy (+1 SD)	−0.07	0.07	−1	0.319
Psychological distress	Low academic self‐efficacy (−1 SD)	0.17	0.07	2.43	0.016
	High academic self‐efficacy (+1 SD)	0.33	0.06	5.5	< 0.001
Cognitive behavior	Low academic self‐efficacy (−1 SD)	0.15	0.06	2.5	0.014
	High academic self‐efficacy (+1 SD)	0.28	0.05	5.6	< 0.001

## 5 Discussion

This study developed a moderated‐mediation model to investigate the effects of academic self‐efficacy, prosocial behavior, psychological distress, and academic stress. The model also aimed to understand how these factors influence each other and the overall relationship between them. The current research shows that while a strong sense of academic self‐efficacy is associated with improved cognitive strategies and increased social involvement, high levels of academic stress weaken students' self‐efficacy and decrease prosocial behaviors. According to the moderated‐mediation analysis, students who have high levels of self‐efficacy are better able to withstand the detrimental effects of stress on their social interactions and thought processes. By focusing specifically on this moderated‐mediation relationship, the research addresses a gap in the literature where scholars have rarely studied the interplay between stress, self‐efficacy, and prosocial behavior in educational settings. In doing so, it clarifies how students with robust self‐belief are better equipped to turn stressful situations into opportunities for reflection and help‐seeking, thereby sustaining collaborative norms even under pressure. Overall, the evidence suggests that self‐efficacy is a promising target for practical interventions, such as workshops designed to teach mastery experiences that can reduce stress, improve learning strategies, and revitalize the caring behaviors typical of resilient academic communities.

Limited research has explored how academic stress connects to psychological distress, especially by differentiating the effects that occur within individuals versus those influenced by interactions with others. As a result, there is limited understanding of the mechanisms or moderators of engagement in this association at the individual level (Kristensen et al. 2023). The present study sought to fill this gap by investigating the role of academic self‐efficacy in shaping the link between academic stress and prosocial actions, using a moderated‐mediation framework. Although scholars have thoroughly documented academic stress and its ties to psychological distress, relatively few investigations have probed whether self‐efficacy softens or strengthens that relationship within learning contexts. Most existing work considers academic stress and psychological distress in isolation, ignoring how cognitive appraisals and the resulting prosocial responses might vary when students believe they can succeed at their studies. Additionally, some studies address how academic stress affects prosocial behavior, particularly when moderated by self‐efficacy. This study fills the gap by examining the moderating role of academic self‐efficacy in the relationship between prosocial behavior and academic stress. Furthermore, identifying the impact of academic stress on prosocial behavior across different academic years, especially different educational stages of students, has not been adequately explored in previous research.

The contribution of insights into how psychological distress, cognitive behavioral, and academic self‐efficacy interact to influence prosocial behavior, addressing the limited exploration of moderation and mediation in this context. Previous studies and the transactional theory of stress and coping have shown a positive relationship between academic stress and psychological distress in students of all educational levels (Lazarus 1984). The likelihood of reporting comparably high or low levels of psychological discomfort at the same time was higher for those who had previously experienced abnormally high or low levels of scholastic stress. Additionally, comparable shifts in academic stress across time were associated with shifts in psychological suffering. Based on these results, interventions are intended to lessen psychological suffering and academic stress (Feiss et al. 2019). The association between students' psychological suffering and academic stress over time was partially explained by prosocial conduct and academic self‐efficacy.

The study found that changes in academic stress were linked to concurrently occurring similar fluctuations in psychological distress and opposing changes in prosocial behavioral patterns and academic self‐efficacy. These findings support the idea that negative emotions in stressful situations can reduce self‐efficacy in those situations (Bandura 1997). Since academic stress is inherently negative, the decreases in self‐efficacy during stressful periods in well‐documented (Usher and Pajares 2008). A key contribution of this study is its demonstration of how changes in academic self‐efficacy can partially account for fluctuations in psychological distress during periods of academic stress. These findings imply that future theoretical frameworks on stress and mental health should consider this mechanism. Especially in research focused on individuals’ experiences. Both the transactional theory (Lazarus 1984) and self‐efficacy theory (Bandura 1997) primarily utilize interpersonal research to understand intraindividual psychological shifts and to elucidate internal processes like cognitive evaluations and emotional responses.

Our results contribute to the current body of research by supporting previous findings that highlight the presence of higher academic stress and lower psychological well‐being among students, as measured using the same two assessment scales (Green et al. 2022; Syed 2021). In contrast, many earlier studies with similar findings examined different dimensions of stress, concentrated on negative mental health effects, or employed diverse methods, scales, and smaller sample sizes. Additionally, many of these studies were conducted in diverse countries, making our research distinct by offering new perspectives on how academic stress interacts with various factors and influences mental health and emotional well‐being (Barbayannis et al. 2022). Our findings indicate that first‐year students experience greater stress and anxiety related to academic performance, which is consistent with the results of previous studies (Lavoie‐Tremblay et al. 2022; Salvarani et al. 2020). Due to the difficulties of adjusting to a new environment and the higher academic obligations faced by first‐year students, the year of study was found to be a major predictor of academic stress (Visier‐Alfonso et al. 2024).

A recent investigation revealed that first‐year nursing students, particularly those lacking any clinical exposure, exhibit heightened academic stress primarily tied to their self‐confidence (Hwang and Kim 2022). The authors argue that early clinical practice cultivates resilience, equipping learners with healthier coping mechanisms to meet the rigors of the program (Salvarani et al. 2020). In contrast, students in higher years say they feel more general stress and academic pressure, which means that the increasing challenges of advanced classes and clinical duties can overshadow the confidence they built earlier (Jimenez et al. 2010). The observed differences may follow from when stress levels are recorded; they typically peak near semester's end, especially among first‐year students who shoulder a heavier theoretical load (Onieva‐Zafra et al. 2020). In addition, the current study shows that academic stress predicts psychological well‐being more strongly than clinical stress, a factor that earlier research had treated as the primary source of stress (Gurková and Zeleníková 2018). Such discrepant findings may also stem from diverse curricula, which have evolved and continue to vary from one country to another.

Approximately one‐third of university students report experiencing high levels of academic stress at various stages of their education, as revealed by this study. A similar study in Nepal reported that around 25% of students experienced high academic stress, which is somewhat lower than the findings of this study. However, research conducted in rural Nepal found similar results (Kaphle et al. 2024). According to a study conducted in Saudi Arabia, 29.6% of pharmacy and medical students had significant levels of academic stress, while a study conducted in Malaysia likewise revealed high levels of academic stress among medical students (Al‐Shagawi et al. 2017; Elias et al. 2011; Gurung et al. 2020). Conversely, a 2010 study in Pakistan reported that 20.8% of medical students experienced high stress, while 71.6% reported moderate stress (Sohail 2013). The discrepancies noted may originate from variations in testing settings and the instruments employed to collect data. Consistent with this, another investigation found roughly one‐third of medical students endured high levels of academic stress, a pattern also observed in the present study (Ruzhenkova et al. 2018; Sohail 2013). Furthermore, earlier work has shown that learners at distinct educational phases report different intensities of stress (Allara et al. 2019). Respondents typically cited heavy assignments, the drive to keep a high CGPA, and the burden of coordinating research as their primary sources of academic anxiety.

## 6 Limitations and Practical Implications

This study fills an important gap in the literature by proposing a moderated‐mediation model that examines how academic stress, academic self‐efficacy, psychological distress, cognitive behavior, and prosocial behavior work together in shaping students. The framework provides a structured overview of how these variables interact and illuminates the student experience as a whole. In keeping with the model, academic stress appears as the primary conduit, shaping mental health, self‐confidence, cognitive patterns, and constructive behaviors both directly and through later stages of the sequence. In contrast, constructive behaviors, persistent prosocial engagement, effective cognitive strategies, and enhanced self‐efficacy correlate with diminished academic stress and stronger psychological resilience. These findings underscore the value of cultivating positive psychological attributes as a means of alleviating academic pressure and, in turn, boosting students' general well‐being. By doing so, the study clarifies the relationship between academic stress, learning, and self‐efficacy, while also offering insights into the mechanisms that drive this connection.

This study has several limitations that future research should address. One key limitation is the use of a cross‐sectional design, which prevented an evaluation of the long‐term stability of the mechanisms. Future studies should adopt a longitudinal approach to assess how these effects persist over time. Additionally, the study employed the transactional theory of stress and coping as a framework and concentrated on factors that were directly related to academic traits (Lazarus 1984), suggesting that factors such as stress, behavioral strategies, learning coping, social support, teacher instruction, and peer relationships may also influence academic stress and psychological distress, so future research should consider these additional variables. Third, studies on teachers have shown that stress hurts their ability to instruct, despite some managerial psychology research suggesting that anxiety may have a favorable association with academic achievement (AbuAlRub 2004; Gao 2023; Jamal 1984). The current study found a linear relationship between academic stress and academic self‐efficacy (Kumari 2020). Future research could explore whether their relationship might be nonlinear. Lastly, the reliance on self‐report questionnaires may have introduced bias due to social desirability, potentially distorting the accuracy of the data. These issues should be addressed in future studies by using alternative methods to gather more reliable data.

## 7 Conclusion

This study highlights a substantial gap in the research concerning the factors and moderating factors examining the effects of academic stress, cognitive behavior, academic self‐efficacy, psychological distress, and prosocial behavior, especially among undergraduate and postgraduate students. This investigation explored how a set of closely related student characteristics and experiences is intertwined. The findings demonstrated that academic stress both directly and indirectly contributes to psychological distress, with self‐efficacy acting as a filter. Importantly, these patterns held stable across a multiyear follow‐up. In addition, elevated distress in turn feeds back into higher levels of academic stress, and this bidirectional loop spills over to shape students' cognitive behavior and prosocial behavior. Across the full sample, moderated‐mediation analyses indicate that self‐efficacy either buffers or amplifies the relationship between stress and mental health: Resilient students appear less harmed by academic stress, whereas those who doubt their abilities suffer more. Moreover, academic self‐efficacy, cognitive strategies, and prosocial behaviors each independently mediate the pathway from academic stress to psychological distress. The evidence points to a reciprocal loop in which stress fuels distress and vice versa, yet this loop is not deterministic; several moderating and mediating variables shape its intensity and direction. Specifically, cognitive strategies and prosocial actions translate stress into distress, while the level of academic self‐efficacy determines how pronounced that translation becomes. These findings underscore the value of targeted interventions that strengthen efficacy beliefs and promote constructive coping, thereby improving the overall well‐being of undergraduate and postgraduate students.

## Author Contributions

**Iftikhar Ahmed Charan**: conceptualization, investigation, writing – original draft, software, methodology, validation, writing – review and editing, data curation, formal analysis. **Wang Chongjin**: conceptualization, investigation, funding acquisition, writing – original draft, writing – review and editing, supervision, resources, project administration. **Shazia Soomro**: writing – original draft, investigation, conceptualization, methodology, visualization, writing – review and editing, software, data curation.

## Ethics Statement

The study received ethical approval from the research ethics committee at Shandong Technology and Business University, China (ref. no. 22BJY025), and complied with ethical guidelines outlined in the Declaration of Helsinki. Before their participation, all individuals were provided with detailed information regarding the purpose of the study, the procedures involved, potential risks and benefits, and their right to withdraw at any time without any consequences.

## Consent

Written consent was obtained from all participants prior to the study.

## Conflicts of Interest

The authors declare no conflicts of interest.

## Peer Review

The peer review history for this article is available at https://publons.com/publon/10.1002/brb3.70907

## Data Availability

The data supporting this study's findings are available from the corresponding author upon reasonable request.
